# Identifying behavioural barriers and facilitators to engaging men in a community-based lifestyle intervention to improve physical and mental health and well-being

**DOI:** 10.1186/s12966-023-01425-1

**Published:** 2023-03-06

**Authors:** Oliver J. Bell, Darren Flynn, Tom Clifford, Daniel West, Emma Stevenson, Leah Avery

**Affiliations:** 1 Population Health Sciences Institute, Faculty of Medical Sciences, Newcastle University, Newcastle Upon Tyne, UK; 2 Newcastle United Foundation, Newcastle Upon Tyne, UK; 3 Department of Nursing, Midwifery and Health, Faculty of Health and Life Sciences, Northumbria University, Newcastle Upon Tyne, UK; 4 School of Sport, Exercise and Health Sciences, Loughborough University, Loughborough, UK; 5 Centre for Rehabilitation, School of Health and Life Sciences, Teesside University, Newcastle Upon Tyne, UK

**Keywords:** Mental health, Physical health, Health behaviour change, Qualitative research

## Abstract

**Background:**

There are few community-based lifestyle interventions designed to target physical and mental health of men. We conducted a qualitative focus group study with men to explore their perceived barriers and facilitators to uptake and engagement with interventions designed to improve their physical and mental health and wellbeing.

**Methods:**

A volunteer sampling approach (advertisements posted on a premier league football club’s social media) was used to recruit men aged 28 to 65 years who were interested in improving their physical and/or mental health and wellbeing. Focus group discussions were conducted at a local premier league football club to 1) explore men’s perceived barriers and facilitators to uptake of community-based interventions; 2) identify health issues considered important to address; 3) obtain participant views on how to best engage men in community-based interventions; and 4) use the findings to inform the development of a multibehavioural complex community-based intervention (called ‘The 12^th^ Man’).

**Results:**

Six focus group discussions were conducted (duration 27 to 57 min) involving 25 participants (median age 41 years, IQR = 21 years). Thematic analyses generated seven themes: ‘Lifestyle behaviours for both mental health and physical health’; ‘work pressures are barriers to engaging with lifestyle behaviour change’; previous injuries are barriers to engagement in physical activity and exercise’; personal and peer group relationships impact on lifestyle behaviour change’; relationships between body image and self-confidence on mastery of skills for physical activity and exercise’; building motivation and personalised goal setting’; and ‘credible individuals increase uptake and continued engagement with lifestyle behaviour change’.

**Conclusions:**

Findings suggest that a multibehavioural community-based lifestyle intervention designed for men should promote parity of esteem between physical and mental health. It should also acknowledge individual needs and preferences, emotions in the context of goal setting and planning, and be delivered by a knowledgeable and credible professional. The findings will inform the development of a multibehavioural complex community-based intervention (‘The 12^th^ Man’).

**Supplementary Information:**

The online version contains supplementary material available at 10.1186/s12966-023-01425-1.

## Background

Increased prevalence of long-term physical and mental health conditions has impacted significantly on life expectancy [1]. In 2016, the leading causes of life years lost due to premature mortality were cardiovascular, respiratory and Alzheimer’s disease, with evidence that men are disproportionately affected [1]. Men are twice as likely to develop ischaemic heart disease and report poorer health than women [1]. Multiple factors contribute the elevation of health risks of men compared with women due to biological causes including cellular responses to stress [2] and body fat distribution. Men are more likely to accumulate visceral fat [3] which is associated with conditions including metabolic syndrome [4], coronary heart disease [5] and ischemic heart disease [5]. Furthermore, research has shown that men often underestimate health risks [6] and express concern about being too thin or weak when losing weight [7]. These beliefs, paired with the reduced likelihood of engaging with healthcare services lead to increased health risks. Importantly, physical, and mental health conditions frequently co-exist with multi-factorial associations and robust evidence for bidirectionality [8–10], therefore it is important to address physical and mental health conditions to reduce risk of morbidity and mortality.

Men are less likely than women to be diagnosed with depression and anxiety, although men are more likely to report lower levels of satisfaction with life, use alcohol and other drugs, and avoid strategies to cope with depression. Additionally, they are more likely to report specific symptoms of depression including irritability, increased loss of control and aggression, and are less likely than women to access psychological therapies [11]. Furthermore, men are more likely to die by suicide than women in every country worldwide [12], although women are more likely to attempt suicide – referred to as the gender paradox [13].

Several barriers exist that can impact negatively on uptake and engagement of men with interventions that target behaviours to improve physical and mental health [14–17], particularly interventions delivered within healthcare settings by healthcare professionals [18]. A possible explanation is that few interventions are developed or tailored exclusively for men, and the way in which these interventions are offered, do not appeal [19]. Intervention developers often do not consider sex-specificity or gender sensitivity during the development stage [20]. Therefore, uptake and meaningful engagement of men is often sub-optimal, and they are considered, in some cases ‘hard to reach’ [21].

In the context of improving physical and mental health, a wealth of evidence supports the use of physical activity and exercise interventions [22]. However, it is important to fully understand the specific requirements and support needs of men, and specifically their barriers and enablers to engagement with lifestyle behaviour change interventions. A 2015 systematic review of lifestyle interventions targeting men identified 12 programmes informed by consultation with men from design through to implementation [20]. Four of these programmes utilised interest in sport and the ‘power of the badge’ as a vehicle to engage men in group-based exercise sessions at local sport clubs. “Banter” in discussions about sensitive health issues including weight gain/loss was considered key to the success of one of these programmes (Football Fans in Training [FFIT]) [23]. The authors of FFIT reported statistically significant increases in self-reported physical activity and weight loss in the intervention group at 12 months follow-up. Participants described how taking part in a programme at a football stadium made them feel “kinda part of it…” and significantly increased acceptability of the programme [24].

The success for FFIT has been replicated worldwide using different sports to engage men [20]. However, there is a paucity of interventions that address both physical and mental health using football as a vehicle for engagement. Given the impact that mental health can have on sedentary behaviour, and the resultant increased risk that sedentary behaviour can have on physical health, there is a pressing need to understand how interventions can be designed to reduce barriers to engaging in physical activity interventions to improve both physical health and mental health in men.

Understanding men’s perceived barriers and facilitators to engaging in community-based lifestyle interventions is critically important to ensure intervention content and mode of delivery is appropriate and adequately addresses their needs and preferences. However, despite the positive impact of the football-informed community-based interventions, there is a dearth of evidence reporting on the active ingredients of these interventions (i.e., key intervention features including mode of delivery, form, content, and duration). As such, the key components of interventions to promote continued engagement following uptake is still unknown. This hinders replicability, faithful delivery, modification to local needs, and optimisation to maximise engagement and outcomes. Furthermore, previously published qualitative studies focus predominantly on physical health rather than mental health, or both. Where studies do report on barriers and facilitators to uptake and ongoing engagement with lifestyle interventions, they often report on practical barriers and facilitators, and place less emphasis on emotional and psychological challenges [25]. Finally, a large proportion of studies focus on the experiences of women, or do not report sex of participants making it difficult to extrapolate the specific barriers and facilitators of men.

To inform the development of a multibehavioural community-based intervention using football as a vehicle for engagement (‘The 12^th^ Man’ Intervention), we conducted a qualitative focus group study with adult men to: 1) explore their perceived barriers and facilitators to uptake of interventions designed to target physical and mental health; 2) identify health issues considered important to address; 3) obtain participant views on how to best engage men in community-based interventions; and 4) use the findings to inform the development of a multibehavioural complex community-based intervention (called ‘The 12^th^ Man’).

## Methods

This study was conducted with reference to the consolidated criteria for reporting qualitative research (COREQ) [26]. Ethical approval was obtained from Newcastle University’s Research Ethics Committee (Ref. 6228/2018). All participants provided informed written consent prior to participation.

### Design

A qualitative focus group study.

### Participants and setting

A volunteer sampling approach was undertaken to recruit participants from the local community using advertisements placed on social media (Twitter, 14,453 followers, May 2018) and Facebook, 108,229 followers, May 2018) accounts associated with the club community organisation Newcastle United Foundation [NUF]). Potential participants were asked to indicate their interest through the social media channel, or by using the contact email address or telephone number provided. Invitations to participate were also sent directly to men who fulfilled the eligibility criteria who had previously registered their interest in community health and wellbeing programmes with NUF. Those indicating their interest were emailed a copy of the participant information sheet and consent form. Eligible participants were men aged between 28 and 65 years who were interested in improving their physical and/or mental health and wellbeing. This age range was selected because 30 and 65 years are two milestones within a man’s life, according to the health, illness, men and masculinities framework [27]. At age 30, men begin to consider establishing a family which can be socially isolating. At age 65, men consider retirement which, again, brings with it social isolation and a loss of purpose. Recruitment took place between May and August 2018. Informed written consent was obtained from all participants prior to the conduct of the study. All consenting participants were screened for eligibility prior to the conduct of focus groups. No incentives were provided for taking part.

### Data collection

Focus group discussions were conducted in person between July and November 2018 in a meeting room located at a Premier League Football stadium, although participants could request an alternative location (e.g., workplace) if this was their preference. Participants were asked to provide their age only so not to create barriers to participation.

An interview topic guide (see Supplementary document) was developed, informed by research literature to facilitate discussion about the following topics: perceived barriers and facilitators to uptake of interventions aimed to improve physical and mental health and wellbeing, saliant health issues, and views on how to best engage men in interventions. A total of 20 questions were asked. The topics were informed by a scoping review of the literature conducted to identify existing lifestyle interventions targeting at men (unpublished). The articles identified were further screened to identify associated qualitative findings reporting on barriers and facilitators to uptake and engagement with lifestyle interventions. Although the topic guide was not pilot tested prior to initial data collection, research team members met following the conduct of focus group one to discuss the data generated and to identify any potential issues with the topic guide. This process was repeated following the conduct of focus group two.

All focus groups were facilitated by one researcher (OJB) who had no previous contact with study participants. The facilitator was a 25 year old male PhD student with an academic background in Sport, Exercise and Nutrition. He wore a premiership football club tracksuit corresponding to the football club where the focus groups were held where he was also employed at the time. The researcher was supervised to conduct focus groups by two members of the research team (LA and DF) who have expertise in qualitative research methods, including the conduct of focus group discussions. No other individuals from the research team were present during focus group discussions.

### Data Analysis

Focus groups were audio recorded, transcribed verbatim and data thematically analysed using inductive thematic analysis [28]. Following the completion of the first focus group, the transcript was read and re-read by two researchers (OJB and LA) who independently applied preliminary codes to the data. The same two researchers subsequently held a meeting to discuss preliminary coding, agree on a coding framework, and any modifications to the topic guide ahead of the second focus group discussion. This process was repeated by the same two researchers following each focus group until it was agreed that the data generated was meaningful and had reached the point of saturation. MS Excel was used to manage the data.

## Results

Six focus group discussions were conducted. Five took part in a meeting room at the Premier League Football club (*n* = 18 participants), and one in a workplace setting (*n* = 7 participants). The duration of group discussions ranged from 27 to 57 min (median time 45 min (inter quartile range [IQR] = 14 min) and involved a total of 25 male participants (median age 41 years, IQR = 21 years).

All questions within the topic guide were asked, although, a minority of questions generated limited or closed responses that did not constitute a theme. Thematic analysis generated seven themes. These are presented in Table 1 with supporting direct quotes and key recommendations for intervention development. Each transcript was coded immediately following the conduct of each focus group. Codes were generated and placed into categories, and categories were assigned preliminary labels. Some themes generated more codes than others. For example, theme 1 ‘Lifestyle behaviours for both mental health and physical health’ and theme 5 ‘Relationship between body image and self-confidence on mastery of skills for physical activity and exercise’ generated more codes than other themes across focus groups. Theme 2 ‘Work pressures are barriers to engaging with lifestyle behaviour change’ was generated from codes from across groups, but mostly from the workplace setting focus group. Initial codes included ‘goals’ that were linked to both mental and physical health, ‘enjoyment’ linked to any activity undertaken to make positive lifestyle changes, and ‘stress’ that was most often associated with work, but also other life pressures including family and inability to access free time.

**Table 1:** Summary of themes identified from transcripts of focus group discussions and key recommendations

Theme	Supporting Quotes	Key recommendations
Lifestyle behaviours for both mental health and physical health	“Good lifestyle, good food, fitness, going to bed at a decent time [all influence physical and mental health]..” (Participant 2, aged 48) “I’ve started going to bed earlier, now, to get the sleep in and I find now I’m getting more sleep and I’m not as stressed” (Participant 5, aged 61) “I think wellbeing is the best word ever because it’s not like you [only] need to be fit, [or that] you need to stop eating bags of chips. It’s wellbeing. I think that is definitely mentally as well as physically.”(Participant 24, aged 51)	• Reinforcement of links between lifestyle behaviours and physical and mental health by a credible individual or credible resource
Work pressures are barriers to engaging with lifestyle behaviour change	“I think most of it is work, as with most people nowadays. Where there’s less people working and they’re expecting people to pick up the work and the workload’s getting heavier…” (Participant 5, aged 61) “You want to do exercise, but if you get up at 05:30 in the morning to get here [work place], and you are getting home at 6:30 pm, you just want to have something to eat, have a shower, and go to bed” (Participant 9, aged 33)	• Provision of workplace interventions and self-help activities • Engagement with employers • Incorporate barrier identification and problem solving activities • Signpost to facilities close by to reduce time to travel • Tackle workload issues practically and personally
Previous injuries are barriers to engagement in physical activity and exercise	“About a year ago I hurt my Achilles and I haven’t been able to run properly since” (Participant 4, aged 38)	• Include graded tasks and facilities/activities to enable this process • Incorporate personalised barrier identification and problem solving
Personal and peer group relationships impact on lifestyle behaviour change	“….if I went and trained and stuff and went away, participated in events and stuff, I don't think she'd be too happy” (Participant 25, aged 39) “…if you’ve got a peer group that’s going to keep you motivated, keep you on target” (Participant 4, aged 38) “…my wife has pushed me on any sport I've done or any fitness thing or healthy eating” (Participant 23, aged 57)	• Incorporate social support when signposting to lifestyle services/programmes/activities. To include social, practical and emotional support
Relationships between body image and self-confidence on mastery of skills for physical activity and exercise	“Whenever you’re big, walking through the door of the gym with all fit and beautiful people is really hard” (Participant 19, aged 45) “There’s no point in me rocking up to a training session that these guys on a Saturday play because I’d just be out of my depth, and I’d look a fool.” (Participant 25, aged 51)	• Generation of rules to address perceived judgement and negativity • Promote group-based activities of similar individuals
Building motivation and personalised goal setting	“But it's having that motivation and having that time when you're not tired to be able to go and take some exercise and say, "Well this is doing me the world of good.” (Participant 16, aged 62) “I think it was small targets that you could see you were achieving, and if you do this you will see results….Setting small targets and seeing small results that help a lot.” (Participant 19, aged 45)	• Identifying a range of options to facilitate lifestyle behaviour change • Identify different ways of making meaningful changes in lifestyle behaviours • Incorporate practical, emotional and social support, and treat all three equally • Offer a means to self-monitor progress
Credible individuals increase uptake and continued engagement with lifestyle behaviour change	“I think, good information, given to you… You’ve got to have somebody there who’s accredited or qualified, to actually tell you what you should be doing” (Participant 3, aged 32) “You look like you’re the type of guy that has got knowledge in these areas [referring to facilitator]. Rather than just doing it myself, I would go to the gym. I’ve got no trainer” (Participant 8, aged 52)	• Use of qualified/trained and non-judgemental individuals who participants can relate to on a personal level (e.g., professionals with lived experience, peer support workers) • Provide up-to-date, evidence-informed information

### THEME 1: Lifestyle behaviours for both mental health and physical health

The consensus from participants was that being healthy involves having good physical and mental health, and both were equally important. Together these increase wellbeing:

*“I think wellbeing is the best word ever because it’s not like you [only] need to be fit, [or that] you need to stop eating bags of chips. It’s wellbeing. I think that is definitely mentally as well as physically.”(Participant 24, aged 51)*

*“I think it’s a healthy mind, so if you have all the stress in the world, that plays a massive part on your health.”(Participant 7, aged 32)*

In addition, positive emotions were reported to be key to optimal health and wellbeing. The importance of being happy and enjoying life was commonly reported across focus groups. Participants often paired happiness with accomplishment of goals and setting themselves a new challenge. When participants were asked to describe the healthiest person they know, happiness was a feature of the response:

*“…they [healthy people] tend to be happier people as well, like [group member] said, they are positive, upbeat and [they] have a target.”(Participant 17, aged 34)*

Another participant supported the view that healthy people enjoy *“…whatever it is that they do.” (Participant 4, aged 38).*

Maintaining a good social life was reported to be instrumental to being healthy and that this could help to cope with stress:

*“But I think having a laugh is the best thing for me and family and friends…” (Participant 17, aged 34).*

The role of a good social life for maintaining health was consistently highlighted by participants:

*“It’s celebrating your success and being happier with yourself and getting a good social life which can help you achieve those goals”. (Participant 6, aged 61).*

*“….sometimes on a Saturday morning we do a health walk.... Sometimes it's not just about the walking, it's the social aspect. It provides health benefits as well. So, it's not just about getting fit, it's about keeping mental awareness.. being healthy, healthy body, healthy brain”* *(Participant 25, aged 39).*

When prompted to discuss the links between lifestyle behaviours, physical and mental health and wellbeing, participants agreed that physical activity and exercise (collectively referred to as exercise by the participants) in particular can have specific benefits for mental health:

*“I think it [exercise] can definitely impact [positively] on your mental health and help you to face the problems that you are facing every day.”* (Participant 6, aged 61).

Participants described the positive effects that exercise had on their lives:

“I’ve found doing more exercise puts me in a better frame of mind, it clears my head”. (Participant 18, aged 34).

“I am getting fitter, again, not just physically but mentally as well”. (Participant 24, aged 59)

One participant explained the importance of his walk home from work and how it allowed him to *“…not bring any stress from here [work] home” (Participant 9, aged 33)*. Others agreed with how physical activity and exercise can impact positively on stress. Some considered it a priority:

*“Fresh air for me, as well. Just a nice walk through the park. So, if it’s been a busy, stressful day, I’ll just walk through the park. It takes an extra twenty minutes to get home but it’s worth it.”* *(Participant 4, aged 38)*

When asked specifically about strategies used to overcome stress, participants referred to specific lifestyle behaviours including sleep, and how sufficient sleep can help prevent or reduce stress levels.

*“I’ve started going to bed earlier, now, to get the sleep in and I find now I’m getting more sleep and I’m not as stressed.” (Participant 5, aged 61)*

This view was supported by other participants across focus groups. However, there were specific lifestyle behaviours such as alcohol consumption that participants were aware had a negative impact on sleep quality and subsequently stress levels:

*“…you have a few drinks to try and de-stress which seems to improve your sleep a bit. Then you start suffering from insomnia and then you drink again, and it becomes a cycle.”* *(Participant 4, aged 38).*

### THEME 2: Work pressures are barriers to engaging with lifestyle behaviour change

This theme was salient throughout all six focus group discussions, however the focus group conducted in the workplace setting generated more frequent work-related barriers and enablers to lifestyle behaviour change. Increasing workloads and ‘doing more and for less’ featured in a large proportion of the participants’ lives. This was linked to long working hours that reduced the time and motivation for engaging in health promoting behaviours:

*“I think most of it will be work, as with most people nowadays. Where there’s less people working and they’re expecting people to pick up the work and the workload’s getting heavier…”* (Participant 5, aged 61).

The increase in workload and lack of time to complete daily tasks was described as ‘frustrating’.

*“If I plan to do something at a certain time and I don't get to do it, I feel as though I'm playing catch up for the rest of the day…” (Participant 15, aged 35).*

The recurrence of this pattern was described to decrease the opportunity to engage in positive health behaviours and reducing or removing this barrier would likely be mutually beneficial to the individual and the workplace.

Participants described time constraints to be a barrier, even outside of work, which left them with little time to engage with physical activity and exercise, and consider other health-related behavioural changes:

*“You want to do exercise, but if you get up at 5:30 in the morning to get here [work place], and you are getting home at 6:30, you just want to have something to eat, have a shower, and go to bed.”(Participant 9, aged 33)*

Other participants described similar situations across focus group discussions:

“It is pretty hard, I find it anyway, to slot a gym session in somewhere along the line”* (Participant 24, aged 51).*

Time taken to travel to facilities to exercise was reported as a further barrier:

“So it is, it's about time and being able to get there, to the place to do it.”* (Participant 25, aged 39).*

Although participants did acknowledge the importance of making time for yourself *“…you’ve got to make time, haven't you? That's probably the thing*”. *(Participant 8, aged 52).*

### THEME 3: Previous injuries are barriers to engagement in physical activity and exercise

Recovering from injuries (acquired recently or in the past) was a common barrier to engaging in physical activity and exercise. Although exercise was considered a ‘good stress reliever’, several participants had discontinued exercise due to injury:

“About a year ago I hurt my Achilles and I haven’t been able to run properly since” (Participant 4, aged 38).

Exercising after injuries was reported to be a challenge due to lack of motivation following time away from exercise. It was particularly difficult for participants to reclaim the time and effort required to re-engage:*“…after about six weeks, after I’d healed, I just couldn’t get myself back into it*.” *(Participant 10, aged 37).* Furthermore, injuries influenced participant’s physical and mental health:

*“A mate of mine has broken his foot, obsessed with running, literally meticulous in his eating and everything. Since he's done his foot, he's never been so depressed. He's been hitting rock bottom because he can't get out and do that exercise”* *(Participant 18, aged 34).*

### THEME 4: Personal and peer group relationships impact on lifestyle behaviour change

Participants reported several challenges associated with making lifestyle behaviour changes to improve their physical and mental health, however the barriers reported became more of a challenge if those around them made lifestyle behaviour change difficult.

*“…having a group of people around you who are not supportive [negatively affects your lifestyle behaviour choices]” (Participant 17, aged 34)*

“You get home and your* lass [female partner/wife] *says,* ‘Do you want a vodka and coke’”* *(Participant 12, aged 51)*

“I lost 24 kilos, something like that. My friends were saying,* ‘Oh, you’ve lost enough weight now; you’ll look ill if you lose any more weight.’* And I thought,* ‘Alright’. And I stopped and that was the worst thing that I ever did”* *(Participant 5, aged 61)*

*“…. if I went and trained and stuff and went away…*, *I don't think she'd [partner be too happy”* *(Participant 25, aged 39).*

Childcare was also an issue when trying to make positive lifestyle changes. Some participants reported feelings of guilt if taking time out for themselves away from their children:

*“I've got a young daughter. I think it would be quite off for me and my wife both to go to the gym together because we'll have to get my daughter looked after and things.”* *(Participant 25, aged 39).*

Several participants reported how taking part in a group-based programme would provide important social support:

*“…team sports like walking football, extensions on that and I think you'd get the social side of that” (Participant 23, aged 57).*

*“I've always known for a fact, if I engage myself in something that was part of a group, that I would be better because I think that's the way I work but I think it's better to work as a team.” (Participant 17, aged 34)*

Participants consistently reported the belief that social support would help to maintain motivation over time:

“Also, if you’ve got a peer group that’s going to keep you motivated, keep you on target”.* (Participant 4, aged 38)*

‘‘…I’d love to get involved in a group, with the support of everybody around, to help change my lifestyle and change my mind-set…” (Participant 7, aged 32)

Previous participation in group based activities where competition was an important motivator was reported as beneficial: “*I think that became a competitive thing with a group of peers and I think that helped” (Participant 21, aged 41)*.

### THEME 5: Relationships between body image and self-confidence on mastery of skills for physical activity and exercise

Participants consistently reported self-confidence issues when attempting to engage in physical activity and exercise, particularly within community settings:

*“Whenever you’re big, walking through the door of the gym with all fit and beautiful people is really hard…you're trying to hide your body away even though you can't, so it's like you don't want to expose yourself to ridicule.”* *(Participant 19, aged 45)*

*“*I think that was the one daunting thing I hated every time I did go to the gym.* There would be buckets of sweat pouring off of me and I'd only been there for ten minutes and there'd be guys doing crunches with their legs up in the air and stuff, completely posing and looking at every mirror.” (Participant 21, aged 41).*

Self-consciousness and body image concerns extended to buying clothing to participate in physical activities and sports:

*“That’s hard. The people are fit. They know what they are doing. And most sports shops don’t cater for big people. So finding clothes that fit is embarrassing and really really difficult.”* *(Participant 19, aged 45)*

Feelings of anxiety relating to ability to master and perform specific skills was also reported:

*“There’s no point in me rocking up to a training session that these guys on a Saturday play because I’d just be out of my depth, and I’d look a fool.” (Participant 24, aged 51)*

There was an expectation that others would be amused by participants inability to master activities: *“…see all these blokes but it’s mostly women who are fit and muscly and I’m thinking they’re laughing at me*.” *(Participant 19, aged 45).’*

### THEME 6: Building motivation and personalised goal setting

A common barrier reported across focus groups was a lack of motivation to even consider making lifestyle changes. This was specifically linked to physical activity and exercise:

*“taking the first step, that’s the hardest one to take” (Participant 3, aged 32).*

*“But it's having that motivation and time when you're not tired to be able to exercise….” (Participant 16, aged 62)*

*“I've always tried losing weight, doing exercise but it's just finding the motivation*…* because I don't particularly like exercise.”* *(Participant 15, aged 35)*

*“…can’t be bothered. That’s the top and bottom of it” (Participant 9, aged 33)*

Although it was acknowledged that taking the first step was the most difficult, and that once a routine was established it became easier: *“The first few weeks is always the hardest and then you get into a routine and then after that you do feel the benefits” (Participant 17, aged 34),* several participants provided insights into their lack of motivation. For example, enjoyment was frequently reported as an issue. Participants understood the health benefits of increased physical activity and exercise, however the lack of enjoyment experienced during specific exercises was reported to prevent participants from living more active lives:

*“…I just don’t particularly enjoy that gym side of things, using the machines etc. it just doesn’t motivate me…” (Participant 20, aged 57)*

There was consensus that setting and reaching behavioural goals (e.g., achieving physical activity targets; reducing alcohol consumption; increasing sleep duration) would lead to positive outcomes (e.g., improved mental health), and that this would be an important motivator to continue. It was emphasised that goals should be graded and achievable:

*“I think it was small targets that you could see you were achieving, and if you do this you will see results….Setting small targets and seeing small results that help a lot.”* (Participant 19, aged 45)

“Rather than just the big picture, small incremental changes. Instead of thinking,* ‘Oh, this programme is going to go for six months or twelve months. What can I do?’ just little daily changes.”* *(Participant 8, aged 52).*

The focus groups also explored how and why participants monitored their health. Although some participants described using body weight scales to monitor health (i.e., a weight within a normal range was an indicator of health), most participants preferred visual cues, specifically clothing, to gauge whether they were becoming unhealthy:

*“The size of my trousers, that's a big one.” (Participant 23, aged 57).*

Others reflected on a time when they were overweight and expressed a desire to not return to a past state:

*“It's seeing the benefit. It's seeing the visual and thinking, "I don't want to get back to what I was and what I felt like" (Participant 18, aged 34).*

Participants described how feedback from physical activity monitoring devices in relation to their goals impacted positively on their behaviour. Specifically, activity trackers highlighted levels of inactivity and prompted behavioural changes:

*“And you can be surprised how few steps I’ll actually do if I just drive to work, go to work, and go back home again, and potter around the house” (Participant 14, aged 56)*

It was also felt that activity trackers can be used positively to create competition with others: “… *both me and the missus have got iPhones and we’re both pretty much mapping, step by step, doing the same route” (Participant 1, aged 35)*. Physical activity monitoring was also described as a prompt to reach a specific goal:

*“..if you’re near your target, at the end of the day, you just randomly walk around the house trying to just make up the steps? Yes” (Participant 3, aged 32)*

*“I do that in the office, mind. Ten minutes before the hour comes, it tells you how many steps you’ve got to, and you’ve got to do 250 every hour.” (Participant 5, aged 61)*

As referred to previously, family members were often considered barriers to making positive lifestyle changes, however they could also be facilitators. Once family members provided their support, they were considered instrumental to success when achieving goals:

*“Definitely, my wife has pushed me on any sport I've done or any fitness thing or healthy eating.” (Participant 25, aged 39)*

*“If I get a Tuesday off she'll say, ‘Come on, you go to the gym while I go swimming…so I'd go but that's because she's dragging me along…” (Participant 15, aged 35)*

### THEME 7: Credible individuals increase uptake and continued engagement with lifestyle behaviour change

A consistent and salient finding across focus groups was that participation in interventions, designed to improve physical and mental health and wellbeing, would be enhanced if delivered by a credible individual who can provide accurate and evidence-based information. Accreditation or relevant qualifications was considered a marker of credibility:

*“You’ve got to have somebody there who’s accredited or qualified, to actually tell you what you should be doing” (Participant 3, aged 32)*

*“You look like you’re the type of guy that has got knowledge in these areas..” (Participant 9, aged 33).*

*“I think there are men who like facts – This is what you’ve got. This is bad for you. Do this. It will put it right. That’s really what we want.” (Participant 13, aged 60)*

Accuracy of health information was a concern, particularly when the information was received from multiple sources, including the media, which can cause confusion:

*“The media complicate things. They don’t know whether you should be eating this or whether you should be eating that, and you get lost at the end.” (Participant 10, aged 37).*

## Discussion

The aim of our study was to identify the behavioural barriers and enablers to engaging men with lifestyle behaviour change interventions targeting physical and mental health.

Three themes identified related specifically to barriers experienced by participants when engaging in lifestyle interventions and have been identified previously by two systematic reviews [29, 30]. For example, work and family pressures; health and physical limitations; a perceived lack of enjoyment, motivation, and time [29, 30]. Similar barriers identified related to forming intentions to make lifestyle changes, particularly in the context of physical activity and exercise when it was perceived as a chore and not enjoyable. As such, the need to provide signposting or access to a range of enjoyable activities is important to promote uptake and continued engagement. This finding is consistent with Self-determination Theory [31] and specifically, intrinsic motivation involving engagement with an activity based on interest, enjoyment, and inherent satisfaction. Teixeira et al., [32] reported how seeking an internal goal, that leads to personal enjoyment can satisfy basic psychological needs for motivation, and ultimately lead to success. Findings from our study support the value of goal setting and goal pursuit as a means of maintaining motivation when making lifestyle behaviour changes. Specifically, the need to set small, tangible, and achievable goals and the benefits of receiving positive feedback and social and emotional support was considered important. Behavioural goal setting has been used successfully in previous men’s health interventions [23, 33–35] and should be considered a candidate for inclusion in future interventions.

Burgess et al., [29] reported potential gaps in knowledge or a lack of awareness about the importance of physical and mental health when adopting healthy behaviours. Our study did not support this finding; indeed, participants showed a good understanding about the importance of physical and mental health, and how the two impact each other, however knowledge and awareness does not naturally lead to behavioural change [36]. Our findings, and those of Burgess et al., [29], suggest that future interventions should continue to highlight the important relationship between lifestyle and optimal physical and mental health, but should place greater emphasis on practical strategies and problem solving to make and sustain behavioural changes (e.g., graded goal setting, social support, problem solving tasks), location of facilities to reduce time travelling and credentials of those delivering them.

In the current study, participants described a lack of self-confidence relating to mastery of skills which prevent engagement in lifestyle behaviours, specifically physical activity and exercise due to fear of embarrassment, and similar findings have been reported by intervention studies [37]. A cross-sectional study investigating barriers to physical activity in men found that those who did not meet physical activity recommendations were more likely lack knowledge, motivation, mastery of skills, and report intimidation or embarrassment. Furthermore, those who reported increased stress levels were also more likely to report intimidation and embarrassment as a key barrier [38]. These behaviours may further explain why men access mental health services following prolonged periods of displaying symptoms and why one in four men will drop out of management regimens [39]. Systematic reviews have also reported negative attitudes of men towards healthcare, that impacts on intention to access services due to traditional beliefs about masculinity and male gender roles (e.g., emotional control, self-reliance, and being successful at any cost) [40]. This highlights the need for interventions that target emotions and aim to support men to develop coping strategies to overcome these challenges. If emotional barriers to lifestyle behaviour change are not addressed alongside other barriers, uptake and long-term engagement will likely remain low. Recommendations based on our findings include the generation of ‘rules’ to address perceived judgement and negativity; promoting group based activities of similar individuals considering perceived ability. These strategies were suggested by participants and have proven successful in previous men’s health interventions [33].

Work related issues and increased stress was a consistent finding of our study. Workplace interventions have emerged to overcome this issue, however there are few interventions that consider the specific barriers faced by men (e.g., pressure to take on overtime at work, and barriers discussed already) [34]. Seaton et al. reported findings from a gender sensitised workplace intervention that aimed to improve self-reported physical activity [35]. The intervention achieved its aims with an increase in walking by 156.5 min/week and was reported to be acceptable by men [35]. A specific finding, consistent with our study was that participants reported that a reduction in workload facilitated physical activity engagement. Interventions to address this issue would likely create opportunities to make and sustain lifestyle behaviour changes.

Our findings also reflect the role of masculinities in the context of health. The issue of work-related issues as barriers to improving lifestyle behaviours could be interpreted as traditional norms of masculinity discouraging men from seeking help for emotional issues [41]. Furthermore, injuries as barriers to re-engaging with previous levels of physical activities / exercise may reflect men’s hyper-competitiveness and a need to strive for physical prowess [42], which men may feel that they can no longer engage with due to injury, which leads to feelings of inadequacy if men identify with these perceptions of masculinity. In addition, issues related to body image and self-confidence with engaging in exercise in community settings was identified in our study, as well as reflecting perceived societal norms about men’s bodies, also reflects the high numbers of men who report experiencing weight stigma. In one study of 1,513 men [43], 40% reported some form weight stigmatisation (most commonly, verbal mistreatment from peers, family members, and strangers).

Our study identified specific facilitators to lifestyle behaviour change to optimise physical and mental health. These included practical support to set personalised and meaningful goals, graded to build confidence, a means to self-monitor progress, and elicit practical and social support from family and peers. A credible individual facilitating and supporting intervention delivery was considered vital, particularly someone who was qualified and could provide evidence-based information and advice in a friendly manner. Furthermore, it was reported to be important that facilitators understood the specific issues related to lifestyle behaviour change for men, were approachable and non-judgemental, and importantly that they were not healthcare professionals. Healthcare professionals often provide information about the benefits of health behaviour change which is widely reported as a mechanism to encourage behaviour change, increase initial uptake of interventions and to promote continued engagement [36–38]. However, for those who do not engage with healthcare services or who feel pressurised by healthcare professionals, it was considered important that information and support is provided by someone who recipients can relate to and get along with.

Our research suggested that social support, including practical support from family members and peers can both positively and negatively influence health-related behaviours, and this finding has been previously reported [29]. However, in our sample this was often linked to work. For example, greater resistance was experienced when trying to find time to take part in leisure time activities and make healthier choices in terms of diet if the working day had been long. Participants reported feeling guilty and explained how partners became resentful. Despite this, participants also described partners and children as being vital when maintaining healthy and physically active lifestyles once they are on-board. Findings also emphasised that participants prefer to be part of a group of similar individuals that creates a sense of identity. This finding corresponds to outcomes reported by the FFIT study that highlighted the role of ‘banter’ and ‘comradery’ as a source of motivation and social support [23].

Social support has also been used effectively as part of interventions to improve the physical activity of men by introducing competition [33, 35, 44–46] and this was an important finding in our study. Participants reported pursuit of goals and meeting personalised targets and referred to healthy competition within group environments. Competition has been used successfully in behaviour change intervention for male participants within workplaces [35, 47], countries outside the UK [46, 48], and within football clubs [23]. As such, social support and competition should be considered key ingredients of interventions targeting lifestyle behaviour change of men.

Longevity as a motivator for behaviour change emerged as a brief discussion point. The importance of longevity to older men (UK retirement age) is highlighted in the Health, Illness, Men and Masculinities (HIMM) framework [49]. Specifically, as men age, they become more aware of their mortality which, when combined with major life events such as retirement, can increase stress [50]. Our findings support this assertion.

Stress was a consistent topic throughout our focus group discussions. Participants regularly talked about a desire to reduce stress and improve quality of sleep to impact positively on daily interactions. However, few intervention studies have considered the impact of sleep on health, specifically those designed for men [51].

Although the findings of the focus groups conducted in a workplace setting versus the football stadium did not differ markedly, data from the workplace setting generated more work-related barriers and enablers, therefore setting in which data is collected should be considered. Furthermore, it was the opinion of the researcher conducting the focus groups that suggestions of enablers were more forthcoming from football stadium participants. A secondary aim of our study was to use the findings to inform the development of a community-based intervention (The 12^th^ Man) targeting improvements in physical and mental health and wellbeing of men. Specifically, identification of target behaviours and key intervention ingredients to engage men and to support them to make personally important changes to their health-related behaviours was a priority. Findings highlighted that physical activity, exercise, alcohol, and sleep to improve mood, function, well-being, and reductions in weight, were important to men, although interestingly diet was not frequently identified or discussed as a target for intervention. With reference to active intervention ingredients, it was agreed that a future intervention should be delivered by a credible individual, or team of individuals who are knowledgeable, can communicate complex and evidence-based health messages simply, and someone who is non-judgemental who participants can relate to and who is considered part of the group. Social pressure and emotional barriers were consistently reported as factors that prevented participants from engaging in health behaviour change. For example, body image and the negative impact it has on mastery of skills and confidence was reported to prevent engagement with physical activity and exercise. Therefore, including strategies within an intervention to reduce or overcome perceived or actual pressure involving social support is important. In terms of optimal duration, there was no data to suggest when an intervention should start and end. Instead participants talked about the need for interventions that provided knowledge, skills, social support, and signposting to local services/activities of interest to enable long-term, sustainable change.

A key strength of this study was the recruitment strategy that was successful in identifying and recruiting male participants who reported a reluctance to engage in health-related interventions offered by healthcare settings. This suggests that the approach taken engaged a different group of men who might be considered as ‘hard to reach’ [21]. This could be attributed to the links with a Premiership football club for the reasons reported previously [24, 27, 52, 53]. Secondly, the interview topic guide and interviewer facilitated discussions around men’s interests initially to understand what prevented them from pursuing their interests rather than something completely new. This approach was successful in identifying barriers and facilitators that could inform the content of a future intervention by incorporating strategies to enable men to participate in activities of choice to improve health and wellbeing.

A potential weakness of the study is that we did not collect health-related data from participants that could characterise the sample. However, a decision was taken not to collect this information so not to dissuade men from participating in a study that is essentially designed to inform intervention development. Lastly, our research focussed on men between the ages of 30 and 65 years in accordance with the rationale provided within our methods, however we acknowledge that younger men also have mental health needs that require further attention in future research.

## Conclusion

The current study was successful in identifying behaviours (specifically, physical activity and exercise and other lifestyle behaviours including sleep and alcohol, albeit to a lesser extent) as targets for intervention that were perceived by men to be important for optimal physical and mental health and well-being, and barriers and facilitators associated with health behaviour change and maintenance. Key active intervention ingredients were also identified as potential mediators to behavioural change (e.g., setting, mode of delivery, intervention content, duration). Findings will be used to inform the development of the 12^th^ Man intervention designed to target lifestyle behaviours to achieve improvements in physical and mental health of men and for delivery through a Club Community Organisation, Newcastle United Foundation.

## Supplementary Information

**Additional file 1.** 

**Additional file 2.**

## Data Availability

We have provided the topic guide used during focus group discussions as a supplementary file. Focus group transcripts can be accessed following individual requests to the corresponding authors.
