# Supplementary material for: Identifying behavioural barriers and facilitators to engaging men in a community-based lifestyle intervention to improve physical and mental health and well-being
Source: Int J Behav Nutr Phys Act. 2023 Mar 6;20:25. doi: 10.1186/s12966-023-01425-1 (PMC9990339; doi:10.1186/s12966-023-01425-1)
Supplement: Supplementary file 1 — Additional file 1. [file 12966_2023_1425_MOESM1_ESM.docx]

**Focus Group Topic Guide**

| 1 | 1. If you were to imagine the healthiest person you know, what is it that they do to make themselves healthy? 2. How likely do you think it is that this person and others like them are stressed? |
| --- | --- |
| 2 | 1. To what extent do you think making healthy lifestyle choices can reduce or prevent stress? |
| 3 | 1. What sort of things increase your stress levels? 2. What sort of things do you believe reduces your stress levels?   **Prompts**: break from work, time with friends/family, exercise, other |
| 4 | 1. What do you think stops people from making positive lifestyle changes that last long term? What gets in the way? |
| 5 | 1. Would you consider taking part in a course that aims to improve your health and wellbeing? 2. If yes, what would be your reasons for wanting to do this?   **Prompts:** family, work   1. If no, what doesn’t appeal to you? |
| 6 | 1. What could your local community do to help you live a healthier life?   **Prompts**: facilities, environment |
| 7 | 1. What could the football club provide that could help you to make positive lifestyle changes? |
| 8 | 1. How would you help other people like you to make positive lifestyle changes? |
| 9 | 1. Has setting up your own exercise or sports club or team ever interested you? 2. If yes, why has it interested you? 3. If no, what doesn’t appeal to you about this idea?   **Prompts**: knowledge about how to do it, resources to set it up, concerns about people attending |
| 10 | 1. A barrier to people attending exercise programme is often working patterns. How would you create a course that is suitable for everyone’s work patterns? 2. What would you need? 3. Who would you need help from? |
| 11 | 1. What sort of things do you do currently to try and improve your health and wellbeing?   **Prompts**: leisure activities; set daily goals, exercise, diet) |
| 12 | 1. Is there anything else we might need if we were to set up a course to help people make positive lifestyle changes? |
